# Learning from the mistakes of others: How female elk (*Cervus elaphus*) adjust behaviour with age to avoid hunters

**DOI:** 10.1371/journal.pone.0178082

**Published:** 2017-06-14

**Authors:** Henrik Thurfjell, Simone Ciuti, Mark S. Boyce

**Affiliations:** 1 Department of Biological Sciences, University of Alberta, Edmonton, Canada; 2 Swedish Species Information Centre, Swedish University of Agricultural Sciences, Uppsala, Sweden; 3 Department of Biometry and Environmental System Analysis, University of Freiburg, Freiburg, Germany; Universita degli Studi di Sassari, ITALY

## Abstract

In animal behaviour, there is a dichotomy between innate behaviours (*e*.*g*., temperament or personality traits) *versus* those behaviours shaped by learning. Innate personality traits are supposedly less evident in animals when confounded by learning acquired with experience through time. Learning might play a key role in the development and adoption of successful anti-predator strategies, and the related adaptation has the potential to make animals that are more experienced less vulnerable to predation. We carried out a study in a system involving a large herbivorous mammal, female elk, *Cervus elaphus*, and their primary predator, *i*.*e*., human hunters. Using fine-scale satellite telemetry relocations, we tested whether differences in behaviour depending on age were due solely to selection pressure imposed by human hunters, meaning that females that were more cautious were more likely to survive and become older. Or whether learning also was involved, meaning that females adjusted their behaviour as they aged. Our results indicated that both human selection and learning contributed to the adoption of more cautious behavioural strategies in older females. Whereas human selection of behavioural traits has been shown in our previous research, we here provide evidence of additive learning processes being responsible for shaping the behaviour of individuals in this population. Female elk are indeed almost invulnerable to human hunters when older than 9–10 y.o., confirming that experience contributes to their survival. Female elk monitored in our study showed individually changing behaviours and clear adaptation as they aged, such as reduced movement rates (decreased likelihood of encountering human hunters), and increased use of secure areas (forest and steeper terrain), especially when close to roads. We also found that elk adjusted behaviours depending on the type of threat (bow and arrow vs. rifle hunters). This fine-tuning by elk to avoid hunters, rather than just becoming more cautious during the hunting season, highlights the behavioural plasticity of this species. Selection on behavioural traits and/or behavioural shifts via learning are an important but often-ignored consequence of human exploitation of wild animals. Such information is a critical component of the effects of human exploitation of wildlife populations with implications for improving their management and conservation.

## Introduction

Animal behaviour is determined by the genetic makeup and the experience of the animal, resulting in a complex configuration of innate and learned behavioural patterns [1–3]. Behaviours can have a genetic basis, these differences may affect fitness and thereby behaviours can evolve by natural selection [1–3]. However, not all behaviours are genetically based, and they can be culturally transmitted or learned through experience. Personality (innate) traits are supposedly less evident in older animals because they may be confounded by learning [4–7] or by social learning [7]. The innate responses of a species are expected to be performed in a uniform and stereotyped fashion [8], whereas learning is arguably a more flexible process that can have different expressions among individuals depending on the learner and the learning conditions. Predators can select certain traits [9, 10], and, at the same time, prey can learn to adopt anti-predator behaviours, such as changes in risk allocation [11], both of which potentially make older, more experienced animals less vulnerable to predation. The strongest anti-predator responses are related to high-risk situations that occur infrequently during a limited time [11], such as during hunting season. As a consequence of these converging selective pressures, favourable behaviours might be more common among older individuals in a population where individuals are under selective pressure or learn from experience [12, 13].

Adaptation to new environmental conditions (e.g., a new predator, increased human disturbance) leads to changes in animal behaviour that can occur very rapidly and involve learning, and hence can be attributed to behavioural plasticity (see the work by Sol and colleagues [12] for an extensive review on the response of a terrestrial vertebrate to urbanized environments). The capacity of individuals to cope with environmental variation might affect the persistence of a population [14]. When a new predator, or a predator with a new hunting method, enters the system, prey then have the chance to learn, evolve or go locally extinct. The role of learning in shaping wildlife responses is a process often mentioned by researchers as a critical aspect of predator-prey and human-wildlife interactions (e.g., [12] and references therein), yet there are few studies that have empirically demonstrated learning responses to novel predators in wild animal populations (but see [15–17] and discussion in [18]).

We studied a system involving human hunters as the main predator of a large herbivore. Humans certainly are not a recent selective force in wildlife, because we have been harvesting wild populations for millennia using an array of weapons and hunting techniques [19]. However, hunting modalities have changed rapidly over the last few decades as recent advances in technology have been introduced (see discussion in [9]). Modern hunters now have high-powered rifles selecting different behaviours compared to a century ago. There is potential for certain wildlife behaviours to be selected more easily by humans, *e*.*g*., active elk can be spotted and targeted by hunters shooting rifles at ranges up to 300 m [9]. Inherited traits in game species may be adapted to both primitive hunters [20] and predators [21], but under harvest pressure by modern hunters, learning and evolutionary adaptation might play a key role in survival.

Here we investigated whether and how female elk (*Cervus elaphus*) learn to adjust their behaviour as they age under human hunting pressure. Female elk are ideal for studying learning in the wild because the hunting pressure on female elk is moderate to low compared to males [9], females have a relatively long life (+20 y.o.), and they are highly gregarious [9]. Many females survive over multiple hunting seasons, they live in groups where some individuals may get shot, and they experience and survive hunting events [22]. This means that female elk have opportunity to adjust their behaviour through learning. Also, the relatively high risk during a limited time caused by hunting should induce strong responses in the behavioural parameters that we can estimate [11]. The likelihood that a female elk will be shot by a hunter decreases markedly with age [9, 23], with female elk becoming almost invulnerable to human hunters when older than 9–10 y.o. [9, 23]. This is arguably not only due to human selection, but also to behavioural strategies learned and adopted by older and more experienced individuals, e.g., reduced movement rates that might decrease the likelihood of being detected by hunters, and use of safer grounds when and where the likelihood of encountering hunters is higher.

Given this scenario, we used an information-theoretic approach [24] to evaluate two alternative hypotheses that we report here in form of alternative research questions along with related predictions.

(i) Are age-related behavioural differences in females driven only by selection by hunters [9, 23]? If only human selection is at work and not learning, then older animals should show more behaviours that reduce the risk of being killed by hunters than younger animals, but individuals should not adjust their behaviour as they age (i.e., no learning). By monitoring individuals for several consecutive years, we can exclude the influence of learning if monitored individuals do not change their behaviour as they age. We used a novel approach by including elk age as a covariate in our models to disentangle human selection on behaviours from learning processes. In our “selection” models driven solely by human selection, we included the age at capture, which is a constant value associated with an individual elk based on its age when captured. If a 4 year-old (y.o.) elk behaves differently from a 10 y.o. elk, for instance, then the selection model allows us to compare the behaviour of the two animals as a function of age difference (older animal *vs* younger animal) but without the learning process (i.e., no individual behavioural shifts as a function of age are allowed by keeping age to a constant value).

(ii) Are age-related behavioural differences in females driven both by human selection and learning? If learning with experience also is involved, then animals should adjust their behaviour as they age, with special regard to those behaviours adopted to avoid human hunters. This second set of models, therefore, includes the true age of the elk as a covariate, which is allowed to change over time.

Our first hypothesis relies on the rationale supported by Ciuti et al. [9] that surviving individuals that are able to age are not a random subset of the population. Selective disappearance of one kind of individual may occur, with for instance individuals with bolder personality being shoot more easily and thus occurring less frequently in older age classes [9]. Found and St. Clair [25] provided an excellent characterization of the bold-shy continuum (innate behaviours) in elk, and Ciuti et al. [9] showed how some of these traits can be selected by human hunters. With our second hypothesis, however, we aim to show whether learning processes also are involved in shaping the behavioural variability of older individuals in this population.

## Methods

### Ethic statement

Our data collection complied with relevant federal laws of Canada and provincial laws of Alberta. Procedures were reviewed and approved by the University of Alberta Animal Care and Use Committee ACUC–Biosciences (Animal care protocol # 536–1003 AR University of Alberta, Edmonton, Canada), by all jurisdictions of the Alberta Government (Permit Numbers: BI-2008-19, RC-06SW-001 and 23181CN), and by Parks Canada (Permit Numbers: WL-2010-7292, WL-2010-5755).

### Study area

The study was conducted over a six-year period in an area of 46,000 km^2^ in south-west Alberta and south-east British Columbia, Canada. This is a diverse landscape, ranging from flat agricultural grasslands in the east, through the foothills to mixed conifer/deciduous forests and mountains in the west. The range of this elk population is an area under multiple jurisdictions administered by the provinces of Alberta and British Columbia. The elk winter range includes both private and provincial (*i*.*e*., public) land of Alberta, whereas elk migratory corridors and summer home ranges are on provincial lands in Alberta (and to a lesser extent in British Columbia). Cattle ranching constituted the dominant land-use on private land. Activities in the public land also included cattle grazing and natural gas extraction as well as extensive recreational use including camping, all-terrain vehicle (ATV) use, hunting, fishing, and hiking [22]. Elk in this region have experienced decades of disturbance by timber harvesting, natural gas extraction, cattle grazing, off-highway vehicle use, and hunting. However, during the last decade, there has been increasing human disturbance from recreational activities and resource extraction resulting in an increase of infrastructure, human activity, road density and traffic volume on roads. Road densities in the area are at levels known to affect elk movements (0.55 km/km2) [26]. In this area, predators include cougars (*Puma concolor*) and wolves (*Canis lupus*) taking approximately 5% of the study animals (among all radio-collared animals), mostly during winter. Grizzly bears (*Ursus arctos*) and black bears (*U*. *americanus*) also pose a predation risk, especially to calves during spring. For an overview on predator distribution, see previous research conducted in the study area [27–29]. However, human hunting is by a wide margin the largest source of elk mortality in this region (at least the 22% of 182 elk monitored by our long-term research program were killed by hunters, 42% for males, see [9] for more details). Hunting was allowed in most of the area used by our study population (see map reported in S1 Fig). The starting date and duration of both bow hunting and rifle seasons varied over time and across Wildlife Management Units (WMUs). Hunting was not allowed in the Waterton Lakes National Park. Bow-hunting season typically took place in September, followed by the rifle season from mid-September to late October. Rifle-hunting season usually ended between late October and late December depending on the Wildlife Management Unit (see S1 Table for details on hunting times and modalities). During this period of the year, elk typically move from summer to winter ranges (from the Rocky Mountains in the west to prairies in the east–see distribution of animals, S1 Fig). In contrast to a rifle hunter, who might shoot effectively from ranges up to 300m, bow hunters usually restrict shots to less than 40m. Bow hunting was responsible for a small fraction of the hunting pressure and harvest during the study period: according to official harvest data, bow hunters harvested on average less than 4% (range 0–28.7% per year and WMU) of the total hunting bag.

### Elk captures and monitoring

We captured elk during 2007–2012 using net guns from a helicopter when soft snow cover was present on the ground to avoid elk injuries during captures. We equipped female elk with GPS radiocollars (Lotek 4400 with drop-offs, Lotek wireless Inc., Ontario, Canada) and released them immediately after. Experienced personnel carried out all captures. A vestibular canine was taken using dental lifters during the capture to assess exact age through cementum analysis (Matson’s Laboratory, MT, USA) (see S2 Table for details on age variation in the monitored sample, i.e., 1–20 y.o.). Data were obtained from 49 females over at least two consecutive hunting seasons with a 2-h fix rate. We used these data to compute step-lengths and test for behavioural adjustments (learning) in movement rates of females across consecutive hunting seasons (see S2 Table for details on sample size and monitoring periods). We chose to subset the data to the period from September to December, i.e., animal behaviour was recorded during the same period of the year but hunting regime varied spatially and temporally (no hunting, bow, rifle). In addition, because we gathered satellite relocations for females over periods longer than 2 years at variable sampling rates (switching from 2-h to 4-h fix rate depending on battery levels), we obtained a most comprehensive dataset covering multiple consecutive years that was used to analyse how individuals selected forest cover and terrain ruggedness across time. A total of 49 female elk contributed relocation data over 2 consecutive years for the step-length data analysis, whereas the same 49 female elk contributed multiple consecutive years of relocation data (min: 2 years, max: 5 years of consecutive monitoring) for the habitat use analyses.

All radiocollars were outfitted with a remote drop-off device programmed to disengage prior to depletion of batteries. If the device failed, elk were recaptured by helicopter using a net-gun to retrieve the collars. All radio-collars deployed to monitor elk in this area were successfully retrieved. We had no fatalities due to capture and re-capture activities from the sample of elk that we monitored.

### Data handling and analysis

We assigned to each GPS relocation the values of three response variables: terrain ruggedness (from digital elevation models with 30 × 30m resolution [9]); forest cover (no forest = 0, forest = 1, based on canopy cover values from 0–100% where presence of canopy = 1) in ArcGIS 10.1 [30]; and step-length–i.e., the distance travelled between consecutive 2-hour relocations, in meters–using Geospatial Modelling Environment [31] combined with R [32]. We chose step-length because it is a well-known proxy for activity and movement rate [33]. We selected rugged terrain and forest cover because we expected elk to adjust their habitat selection when they aimed to reduce the likelihood of being detected by humans [9, 34–36]. Only positions 2-hours apart during the first 2 years of monitoring for each radiocollared elk were used in the calculation of step-lengths (see S2 Table), because sampling rate decreased after the second year of monitoring, as noted above. The database used to model step-lengths eventually included 2 consecutive years of monitoring per animal, whereas the datasets used to model the use of terrain ruggedness and forest included multiple consecutive years of monitoring per animal (range of consecutive monitoring within the elk sample: 2–5 years).

We modelled variation of three response variables (step-length, terrain ruggedness and use of forest for used pixels) as a function of time of the year (months: Sept.-Dec.), canopy cover (in percentage, in those models where the use of forest was not the response variable) and terrain ruggedness (in those models where use of terrain ruggedness was not the response). We included a quadratic term for the use of terrain ruggedness and canopy cover when used as predictors to account for non-linear effects. We also included proxies of human activities: such as the time of day (dawn, day, dusk and night), distance to roads (close, d<500m; far d>500m; distance based on previous work in the study area [22, 28, 29]), week period (proxy for weekly activity, i.e., weekend or weekday), and hunting season (no hunting, bow, rifle season). We chose those variables because they correspond well to mortality risk during hunting season [9]. Our choice of cut-off distance (500m), chosen based on previous studies [22, 28, 29, 37], assumes that there should be no road effect beyond 500m. More specifically, it was our intention to investigate elk behaviour when the distance to the road matters (e.g., within 500 m *sensu* [22]) compared to when it does not matter (e.g., we did not expect to record a different behaviour between elk when located at 1km, 4 km, or 10 km from the closest road). Previous research conducted on our target population [9] found higher movement rates by elk that were eventually shot by hunters (increased encounters with humans). Ciuti et al. [9] also showed that mortality risk increased for elk moving faster when and where hunter activity was higher (flatter terrain, open areas, close to roads, and only slightly during weekends). Higher movement rates were usually observed at dawn and dusk as a result of crepuscular activity, which correspond to the period of higher hunting pressure.

We modelled step-length (log transformed to achieve normally distributed residuals) using a linear mixed model (LMM) as a function of environmental variables and human-activity-related variables, and the interaction between age and human-activity-related variables. Likewise, we modelled variation in the use of terrain ruggedness using LMM as a function of environmental variables (same as for step-length excluding ruggedness), human-activity variables, and the interaction between age and human-activity variables. Finally, to examine the use of cover by elk, we used logistic regression to model use of forest (0 = no forest, 1 = forest) as a function of environmental variables (same as for step-length, excluding canopy cover as predictor), human activity variables, and the interaction between age and the human-activity variables. Female home ranges were stable and overlapping across years, with over 90% of the home range (minimum convex polygon) of one year included in the next year’s home range. Each elk had the same habitat availability throughout the monitoring period. Thus, variation in the use of terrain ruggedness and forest across years can be assumed to be proportional to variation in selection, because availability was fairly constant through time. We were more interested in the actual use (and change in use over time) of a resource rather than its selection, because we expected that the use of a given resource would be more tied to mortality risk than its selection. A resource unit might be strongly selected by one animal, e.g., when 10% of relocations are located within open areas that were only 1% of the available resources, However, a resource might be weakly selected by a second animal when 50% of relocations were located within open areas that amounted to 49% of the available resources. The latter animal would appear to show weaker selection for open areas (use/availability) but spends more time in open areas than the former animal thereby exposing it to higher mortality risk (sensu [9]).

Migration might coincide with our treatment types (e.g., rifle hunting) and reduced step length could be a result of migratory strategies combined with age and individual behaviour [25, 38, 39]. However, 93% of females monitored with satellite telemetry and included in our dataset were migratory animals (mean autumn migration length ± SE: 21.3 ± 2.0 days; mean linear displacement: 18.0 ± 0.7 km). Little variability in migratory strategies in our monitored sample made our sampling design less vulnerable to noise due to different movement rates usually shown by dispersers and resident elk (see [37]).

### Human selection or learning at work?

To evaluate our alternative hypotheses, we compared models with different ways to account for elk age (age at capture kept constant across years, or actual age recorded in a given year, *i*.*e*., true age). See Table 1 for a complete overview. With true age included in the models, we were able to detect changes in behaviour resulting from experience (*i*.*e*., behaviour changes due to learning). In contrast, if age at capture were used, then within-individual behavioural adjustments due to aging were not considered (i.e., no learning because age was kept to a constant value corresponding to that recorded at capture), and the model investigates the difference among individuals of different ages. In practice, when age at capture was included in the model structure, then we could use the model to detect selection (behaviour of surviving older individuals differing from younger ones). When true age was included in the model structure, the model did not allow us to exclude human selection but we could detect learning (i.e., behaviour of each individual changed as the individual got older).

**Table 1:** Set of generalized linear mixed effect models (Restricted Estimate of Maximum Likelihood) with different random structures and different measures of elk age, either allowing individuals to change behaviour between years or not. Elk have been monitored for multiple years, and the terminology ‘true age’ implies the actual age of the elk in a given year. The term ‘age at capture’ implies the age of the elk kept constant to that recorded at the beginning of the monitored period. ‘True age’ allows models to account for behavioural adjustments with age (learning), while ‘age at capture’ does not allow depicting learning processes. The 5 *a priori models* were run to explain the variability of three different response variables (log step-length, use of terrain ruggedness, use of forest). The top ranked structure (#5) selected using AIC was the same for all response variables. Because model selection was performed on models with different random effect structures, we opted to use the number of levels of the random effects minus 1 as the punishment for added random effects when calculating the AIC.

#	Random intercept for elk identity (ID) and random slope for true age^X^	Random intercept for year of study	Elk age estimate included in the model	ΔAIC^a^ [response variable: log step-length]	ΔAIC^b^ [response variable: use of terrain ruggedness]	ΔAIC^c^ [response variable: use of forest (0 = no forest, 1 = forest)]	Model details	Model key word
1	None	(1|year)	True age	6075.6	28966.2	11248.7	No random effect for individual elk, age allowed to vary	Learning and selection at work, no individuality
2	None	(1|year)	Age at capture	6160.9	28966.8	11160.6	No random effect for individual elk, age not allowed to vary	Only selection at work, no individuality
3	(1|ID)	(1|year)	True age	2880.8	2184.7	4147.8	Animals can change behaviour (learning) between years, but they all learn in the same way (same slope).	Learning and selection at work
4	(1|ID)	(1|year)	Age at capture	2882.1	2115.1	4124.6	Animals cannot change their behaviour (no learning) between years.	Only selection at work
5	(True Age|ID)	(1|year)	True age	0	0	0	Individual animals can change behaviour (learning) as they age	Both individual learning and selection at work

^X^A model with age at capture as random slope is not within the alternative models as such age metric does not change over time.

^a^Fixed effects in the model: month + canopy cover + canopy cover^2 + terrain ruggedness + terrain ruggedness^2 + age*day of the week + age*time of the day + age*distance to road + age * hunting season.

^b^Fixed effects in the model: month + canopy cover + canopy cover^2 + age*day of the week + age*time of the day + age*distance to road + age * hunting season

^c^Fixed effects in the model: month + terrain ruggedness + terrain ruggedness^2 + age*day of the week + age*time of the day + age*distance to road + age * hunting season

We formulated the full models with either age at capture or true age as the fixed effect “age.” We had different formulations of the random effects (Table 1): random intercepts per year of study, random intercepts per individual, and random slopes for true age per individual (see Table 1 for the five different model structures depending on varying combination of random intercepts and slopes). Random intercepts per year only meant that annual differences were taken into account (inter-annual variability due to environmental factors not included in the model structure), and thus they were included in all models. Models with random intercepts per individual allowed animals to differ in behaviour, but not over time. Models with random intercept for individuals and true age as random slope allowed us to model a behavioural shift as a function of age, thus allowing both learning and human selection. See Table 1 for the full specification of model structures. Models were compared using Akaike Information Criterion (AIC) and selected based on the lowest AIC. Because we focused model selection on models with different random effect structures we opted to use the number of levels of the random effects -1 as the punishment for added random effects (*sensu* [40]) when calculating the AIC instead of the less conservative (conditional) cAIC [41].

We fitted the 5 alternative linear mixed-effect models using the Restricted Estimate of Maximum Likelihood (REML) method in R [32] using the packages *lme4* [42]. For the model with the use of forest (binary output) as response, we fitted a generalized linear mixed effect model with binomial distribution of errors. Because we included time of the day as predictor in our models, any trace of temporal autocorrelation disappeared in model residuals [43].

### What behaviours change as animals become older?

After estimating the random structure and age measure we continued by building an *a priori* set of models that we fitted by Maximum Likelihood (ML). Each model differed only by the inclusion of true age and the two-way interactions between true age and human activity measures as fixed effects. This enabled us to verify whether elk behaved differently towards proxies of human activity as they grew older (Table 2). Two-way interactions were generated considering that movement rate (step-length) and habitat use (terrain ruggedness and forest) is expected to change in older individuals depending on the time of the day, distance to road, hunting season, day of the week. This was based on our main expectation that older individuals would move less and would use safer habitat where and when the likelihood of encountering human hunters was reduced (sensu [9]). We calculated AICc in the standard way where the punishment term for the random effects was 1 per random effect as we focused on the fixed effects [44]. Note that we did not change which fixed effects were included in the model (except age) because we were interested in evaluating how age interacts with proxies of human activity. We averaged the models with a total sum of weight of at least 0.90 [24] using the MuMIn package in R [45]. For the fitted models with the lowest AICc’s, variograms of the residuals were plotted to assess if there was spatial autocorrelation remaining after accounting for environmental covariates (S2 Fig).

**Table 2:** Comparison of three sets (1 = log step-length, 2 = use of terrain ruggedness, 3 = use of forest by female elk as response variables, respectively) of Generalized Linear Mixed Models. The structure of the fixed component of the model was constant across models (see Table 1 footnotes) with the only exception of age (not included, included) and age interacted with human-activity proxies (time of the day, distance from road, hunting season, and time of the week). All models had a random slope for true age and a random intercept for individual elk, as well as a random intercept for year–i.e., the best random effect structure selected in Table 1 –and were fit with Maximum-Likelihood estimation. Models indicated by an asterisk accounted for more than 0.90 of the Akaike weights and were further inspected for model averaging (S3 Table).

Fixed effects	ΔAICc^1^	ΔAICc^2^	ΔAICc^3^
Age not included as fixed effect in the model	12.87	382.93	171.53
Age included as fixed effect without interactions	14.72	384.60	174.55
Age included as fixed effect and interacting with:			
Time of day	19.63	376.18	136.99
Dist to Road	14.98	255.35	32.32
Hunting season	0 *	139.75	154.78
Day of week	16.71	384.79	167.07
Dist to Road and Time of day	20.02	240.28	3.01 *
Hunting-season and Time of day	5.00	129.15	137.01
Hunting-season and Dist to Road	0.49 *	17.48	28.70
Day of week and Time of day	21.62	376.39	154.80
Day of week and Dist to Road	16.97	255.54	54.44
Day of week, Hunting-season	1.99 *	140.15	156.46
Hunting-season, Dist to Road and Time of day	5.60	0 *	20.30
Day of week, Dist to Road and Time of day	22.02	240.49	3.44 *
Day of week, Hunting-season and Time of day	6.99	129.57	162.83
Day of week, Hunting-season and Dist to Road	2.48 *	17.88	29.30
Day of week, Hunting-season, Dist to Road and Time of day	7.60	0.42 *	0 *

^1^response variable: log step-length.

^2^response variable: use of terrain ruggedness.

^3^response variable: use of forest (0 = no forest, 1 = forest).

## Results

### Human selection or learning?

We reported in Table 1 five alternative models with different random-effect structures fitted to explain variability in three response variables: log step-length (our proxy for activity and movement rate), use of terrain ruggedness (proxy for the use of steeper and safer terrains), and use of forest (affording cover from hunters). We selected the same best random structure (#5, Table 1) for all response variables, which included year as a random intercept, individual elk as a random intercept, and true age as a random slope. This structure allowed us to model individuals changing behaviour as they age, implying that both learning and selection shape this hunter-elk predator-prey system. More specifically, individual elk adjusted behaviours (movement rate and use of rugged terrain and forest) as they grew older, implying learning (individuals adjusted behaviours as they got older) and, at the same time, human selection (surviving older individuals behaved differently than younger ones). We did not find empirical support for alternative models that did not allow for individual learning (Table 1), thus excluding that selection is the only pressure at work in shaping behaviours in this population.

### What behaviours change as animals become older?

**Fig 1:**
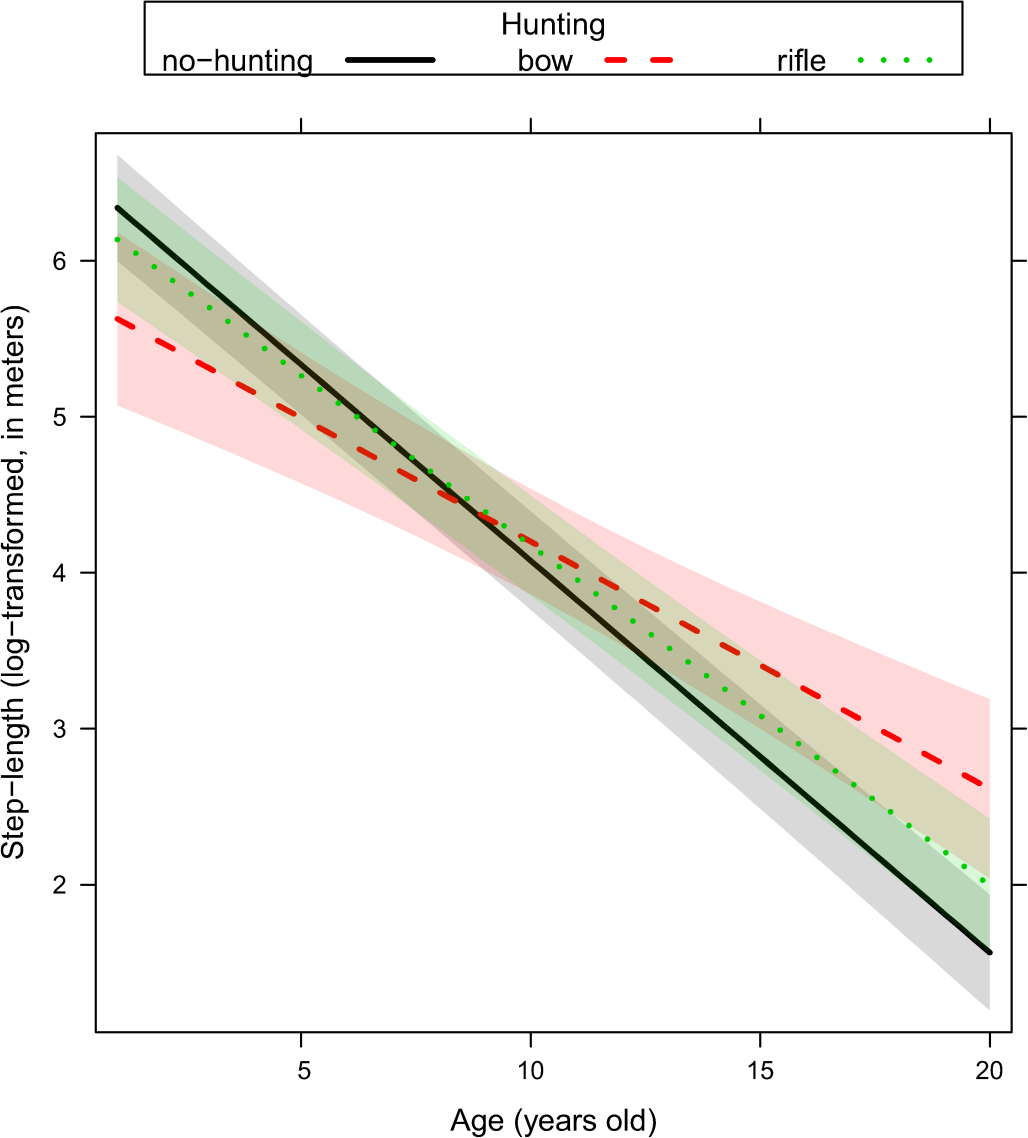
Movement rate (step-length, i.e., distance in meters travelled every 2 hours, log-transformed) in female elk as a function of age (range 1–20 years old) and hunting regime (no-hunting, bow, and rifle) as predicted by the linear mixed effect model. Ninety-five percent marginal confidence intervals are shown as shaded areas [sample size: n = 49 female elk, each of them contributing with telemetry relocations collected over 2 consecutive years].

**Fig 2:**
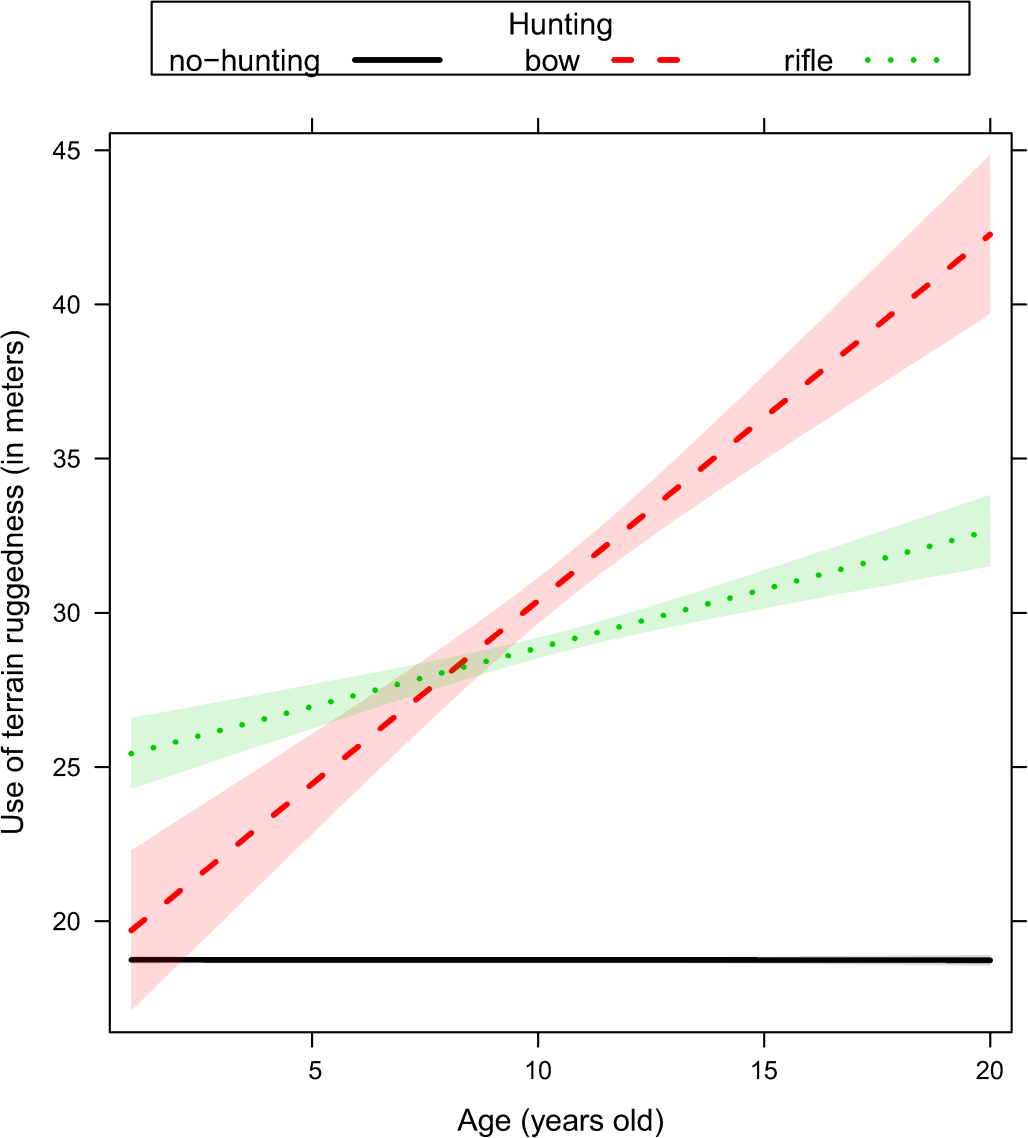
Use of terrain ruggedness (in meters) in female elk as a function of age (range 1–20 years old) and hunting regime (no-hunting, bow, and rifle) as predicted by the linear mixed effect model. Ninety-five percent marginal confidence intervals are shown as shaded areas [sample size: n = 49 female elk, each of them contributing with telemetry relocations collected over 2 to 5 consecutive years].

**Fig 3:**
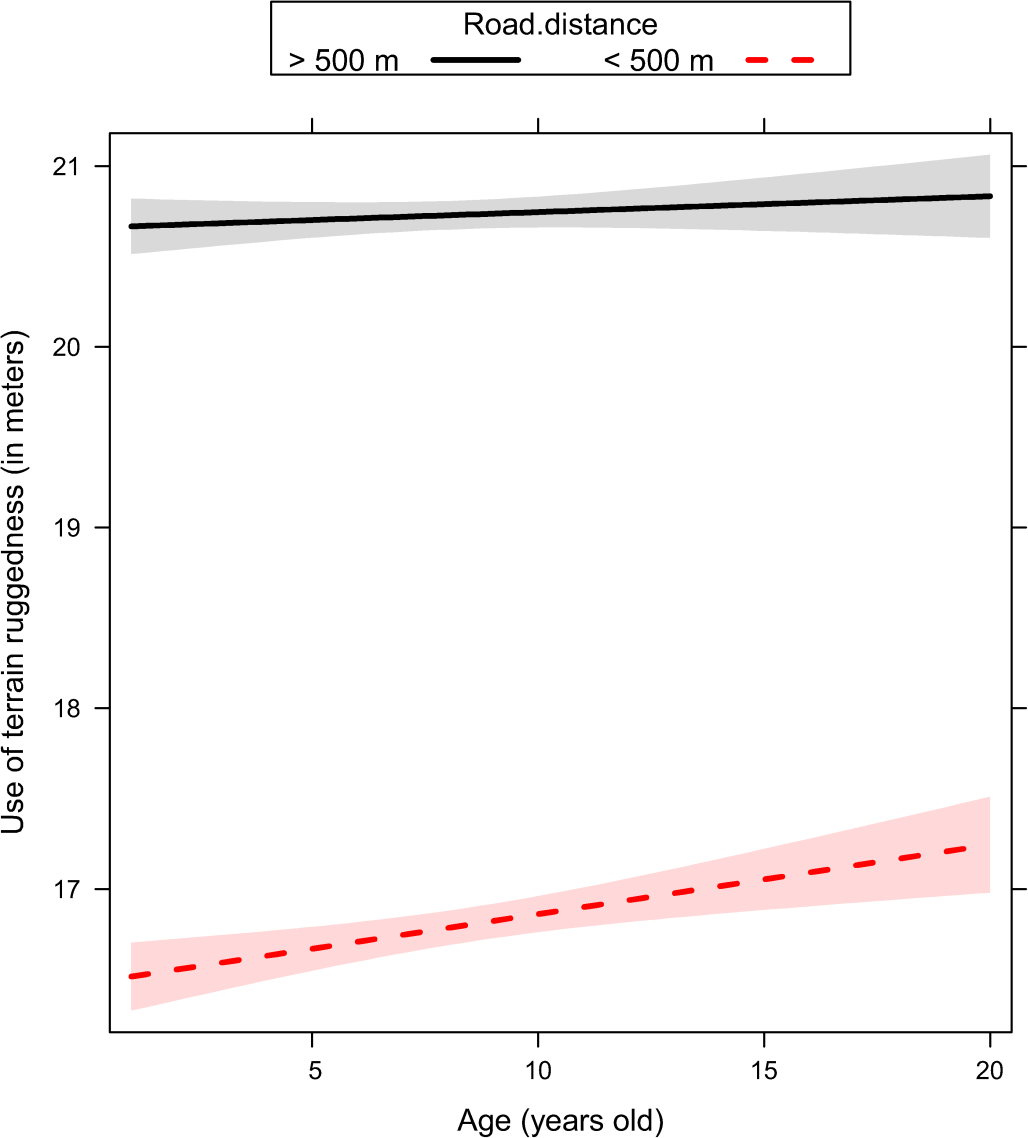
Use of terrain ruggedness (in meters) in female elk as a function of age (range 1–20 years old) and distance to road (distance higher or lower than 500 meters) as predicted by the linear mixed effect model. Ninety-five percent marginal confidence intervals are shown as shaded areas [sample size: n = 49 female elk, each of them contributing with telemetry relocations collected over 2 to 5 consecutive years].

**Fig 4:**
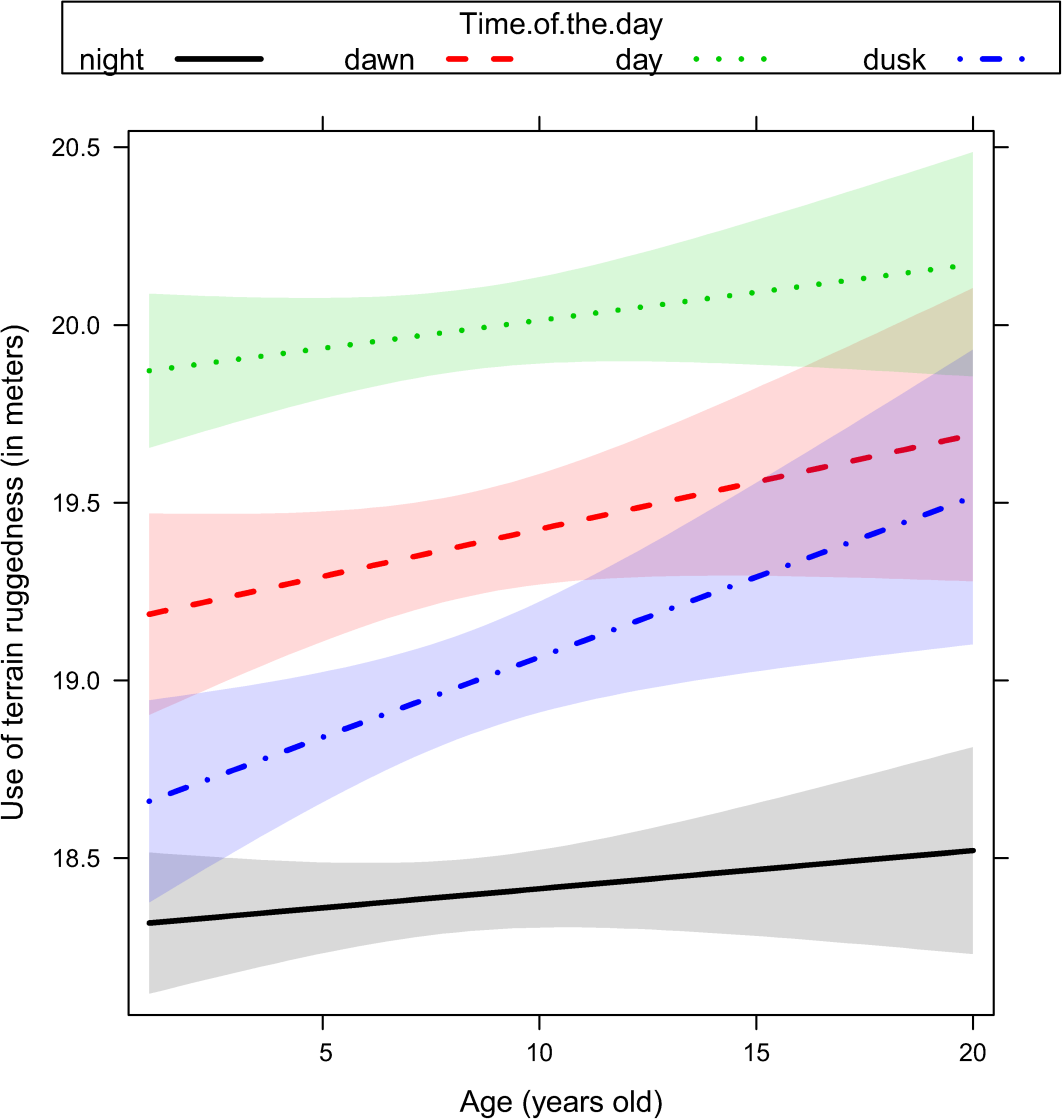
Use of terrain ruggedness (in meters) in female elk as a function of age (range 1–20 years old) and time of the day (night, dawn, day, and dusk) as predicted by the linear mixed effect model. Ninety-five percent marginal confidence intervals are shown as shaded areas [sample size: n = 49 female elk, each of them contributing with telemetry relocations collected over 2 to 5 consecutive years].

**Fig 5:**
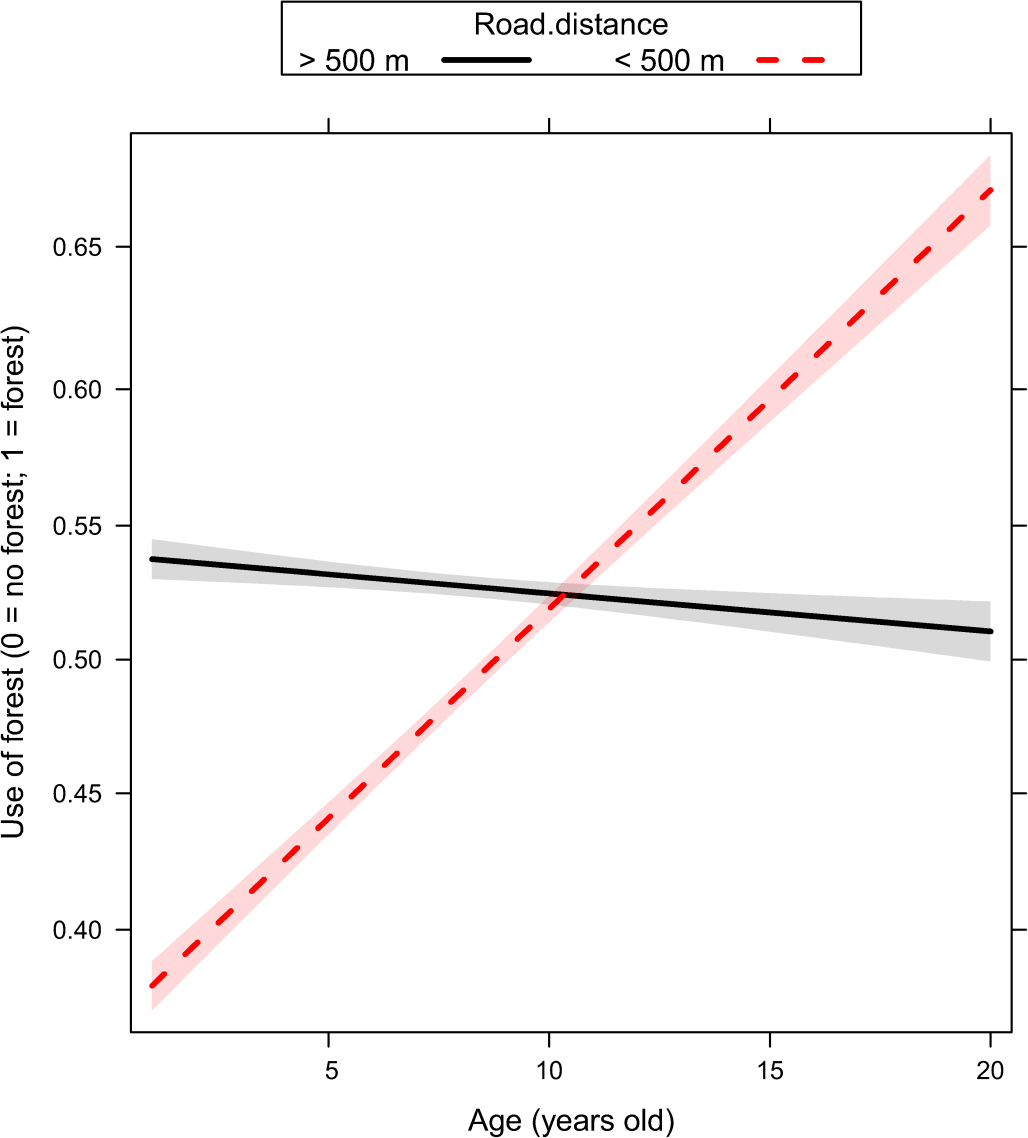
Use of forest (0 = no forest, 1 = forest) in female elk as a function of age (range 1–20 years old) and distance to road (distance higher or lower than 500 meters) as predicted by the generalized linear mixed effect model. Ninety-five percent marginal confidence intervals are shown as shaded areas [sample size: n = 49 female elk, each of them contributing with telemetry relocations collected over 2 to 5 consecutive years].

We ranked alternative models with the same random structure (random structure #5, Table 1) but with a different way to account for age, either excluding it as predictor or including it as single effect or interacted with proxies of human activity (Table 2). Age played a key role in all sets of models fitted to explain the variability of response variables (Table 2). Movement rate and the use of terrain ruggedness and forest were adjusted by older individuals (Table 2) depending on the ongoing hunting regime (no-hunting, bow, rifle), the distance to the closest road (< 500m or > 500 m), and the time of the day (dawn, day, dusk, and night). Given that top-ranked models reported in Table 2 did not receive full support (with a number of models within ΔAICc < 4), we performed model averaging on a comprehensive subset of models with a cumulative Akaike weights higher than 0.90 (S3 Table). In general, female elk showed a reduction of their movement rate (Fig 1, S3 Table) as they became older. Older females increased their use of rugged terrain during the hunting season, and this was recorded to a greater extent during the bow season than during the rifle season (Fig 2, S3 Table). Older females also increased their use of rugged terrain close to roads (Fig 3, S3 Table) especially during dawn and dusk (Fig 4, S3 Table). Finally, females generally decreased their use of forest as they became older, except when they were close to roads, where they increased the use of forest (Fig 5, S3 Table).

## Discussion

Our first hypothesis that age-related behavioural differences in female elk are only driven by selection of behaviours by hunters was not supported by data. Human selection (*sensu* [9]) is not the only pressure at work in our system. We showed that learning processes also play a role in shaping the predator avoidance behaviour in this population. We thus supported our second hypothesis that age-related behavioural differences in females are driven by both human selection and learning.

Female elk in our study population adjusted their behaviour as they became older, adopting behaviours to avoid human hunters. Despite extensive wilderness in our study area compared to more heavily human dominated landscapes, effects of humans on behaviour of wildlife exceed those of natural predators in this landscape of fear [22]. Hunting is the largest mortality risk for elk, with roughly 50% of males and 20% of females monitored by our long-term research program shot by hunters, but less than 5% killed by cougars or wolves [9, 22, 27].

By comparing alternative random structures in our mixed-effect models, we showed that individual elk adjusted their behaviour (movement rate and use of rugged terrain and forest) as they grew older, due to both learning and selection. Movement rate and the use of terrain ruggedness and forest were adjusted by older individuals depending on the ongoing hunting regime (no-hunting, bow, rifle), proximity to a road, and as a function of the time of the day (dawn, day, dusk, and night). Our study highlights elk behavioural plasticity to hunting pressure, confirming patterns in habitat selection previously documented by Proffitt and colleagues [46]. Female elk reduced movement rates as they aged, which was linked to reduced detectability and likelihood of encountering human hunters [9, 47]. Older females increased the use of rugged terrain during the hunting season to a greater extent during the bow compared to the rifle season. Steeper terrain is commonly considered safe ground for ungulates [48, 49], but the stronger effect recorded during the bow season might be related to different antipredator strategies adopted by elk to avoid bow hunters compared to rifle hunters [50–52]. Rifle hunters can arguably shoot into rugged terrain from a longer distance because target visibility should be more favourable on slopes than in flatter terrains. In contrast, stalking close enough for a bow hunter might be more difficult in more rugged terrain, and hunters would be more easily detected. Also arguably elk could be more adapted to that type of behaviour by hunters because bow hunting is an older tradition (millennia) compared to hunting with high-powered rifles (decades). On one hand, rifle hunting is expected to have the largest effects due to the direct threat to the animals [9, 47]. On the other hand, bow hunters likely elicit different behavioural response because the hunter must get much closer to the animal. The end result is that both hunting techniques evoke a behavioural response in elk, and elk differentiate between the different hunting seasons. With age, females increase their use of rugged terrain close to roads. Females generally decrease their use of forest cover as they become older, although they significantly increase the use of forest when close to roads, exactly where and when the likelihood of being spotted by hunters would be highest. Thus, elk learn to differentiate risks associated with roads compared to areas farther from roads. All these results are in agreement with the strategy adopted by this population to successfully avoid human hunters [9].

Innate responses by a species are expected to be performed in stereotypic fashion [18], whereas learning is arguably a more flexible process and can have different outcomes depending on the learner and the conditions for learning [53]. This result has been shown to apply to other species in very different ecological contexts, such as guppies *Poecilia reticulata* [53] and wallabies *Macropus eugenii* [54]. Learning shapes the behaviour of female elk, and learning is a highly individual process. Our results showed that it is important to let individuality emerge when making population-level inferences. Recent studies targeting different species and ecological contexts are increasingly paying more attention on the importance of considering inter-individual variability as a key component of a population rather than noise in the analyses [9, 25, 55–58]. Large variation in the learning process may be due to variation in the factors affecting the animal when and how learning occurs. Elk vary considerably in how they change their overall behaviour as they age. However, when it comes to their response to hunters, they change their behaviours in a uniform way (decreased movement rate, increased use of forest and rugged terrain). Even though these responses may vary in magnitude among animals, they all go in the same direction, which is typical of strong anti-predator responses that are related to high-risk situations that occur infrequently during a limited time [11], such as during hunting season.

Learning about mortality risks should be more efficient in gregarious species (such as for elk aggregations) due to opportunities for non-lethal exposure to mortality, [e.g. 59–62]. Learning about predators can occur through direct experience or through social learning from experienced individuals. Social learning provides individuals with an efficient means of obtaining information while reducing the costs associated with direct learning. An example is provided by the social learning by coral reef fishes, *Pomacentrus amboinensis*, that highlights how information on predator identities can be passed to group members quickly without a dilution of information content [59]. Gregarious animals with similar cognitive abilities compared to elk—such as reindeer *Rangifer tarandus* habituated to humans [63]—are able to learn about novel predators as they grow older [15–17], whereas solitary animals such as gerbils *Gerbillus andersoni allenbyi* do not appear to have that ability [64].

The perception of risk might differ according to the individual personality type, because animals might adjust their behaviour differently according to their behavioural type. See Bonnot et al. [57] for an example in roe deer *Capreolus capreolus*. The consequence of inter-individual differences in learning can lead to the ability for an animal to learn and to adapt its behaviour as a function of its behavioural type, *i*.*e*., are shyer individuals more plastic? See Kareklas et al [56] for an example in fishes. Because the probability of being shot is increasingly thought to depend on personality type [6], we might expect that individuals with a certain personality type could adapt their behaviour more quickly by learning. Our mixed models were flexible enough to allow learning processes to be depicted as a function of the age (*i*.*e*., random slope for age and random intercept for individual, thus allowing animals of different age to learn differently). Further investigations should identify personality traits from spatial behaviour [55], and then disentangle how learning specifically varies as a function of personality types. There is an urgent need to deepen our understanding of the relation between inter-individual variability in behaviour in the wild to personality traits [58] to better tackle variation in the responses by wildlife to human activity.

As introduced earlier, human hunting pressure on elk is much higher on males than females. This means that the likelihood for certain behavioural traits selected by humans (namely bold individuals) to occur is higher among older females compared to older males. On the one hand there is a strong selection by hunters on male behavioural traits [9] where”bold” individuals are more likely to get shot, and high hunting mortality limits the chances for bold individuals to survive and learn how to cope with hunters. On the other hand, however, females have more chances to learn how to deal with hunters because of reduced hunting pressure, with higher likelihood of survival for bold females because they have more chances to learn. This could sustain some behavioural traits in the population such as boldness or aggressiveness, at least among females, despite being selected against by hunters, which could guarantee the inter-individual variability that is key to population resilience against contrasting selection pressures [65–68], such as wolf predation [23, 69].

The likelihood that a female elk will be shot by a hunter decreases markedly with age [9, 23], with female elk becoming almost invulnerable to human hunters when older than 9–10 y.o [9, 23]. Our study contributes to a better understanding of this phenomenon, because the behaviour of surviving animals is shaped by both human selection and learning. This, however, introduces contrasting selective pressures in the elk predator-prey system when comparing selection by human hunters and natural predators. Other predators such as wolves, cougars and bears are also expected to impose selective force on elk behaviours. In a long-term study in the Yellowstone ecosystem, Wright and colleagues [23] showed that the age classes of female elk selected by wolves and hunters were significantly different. Hunters selected a large proportion of female elk with the greatest reproductive values (mean age: 6.5 y.o.), whereas wolves selected elk calves and older females with low reproductive values (mean age: 13.9 y.o., [23]). Because wolf and hunter techniques differ, and they arguably select for different behavioural types, management of wildlife populations should avoid hunting pressures that might remove behavioural traits that could help animals to cope with natural predators. Bold females are more likely to survive than bold males in our study system, and this could maintain enough individual variability to allow elk to cope with native predators like the wolf (see discussion in [69]). Innate behavioural traits are arguably more important for coping with natural predators as well as human hunters using traditional hunting techniques (e.g., bow), whereas learning could play an important role where animals have had not enough evolutionary time to adapt (e.g., rifle hunting). Both voles and rabbits seem to eventually adjust to novel predators (e.g., learning), even if they have stronger responses to predators with which they have co-evolved [70, 71].

We showed that learning can have a long-term effect on the behaviour of individuals in a wild population. We also showed that animals were able to differentiate between hunters using different equipment and tactics and to fine-tune their behaviours accordingly. This helps to explain why other indices of human activity such as roads can have vastly different effects on behaviour of large mammals inside and outside of national parks. For example, animals inside parks without hunting do not avoid roads like they do outside parks [72]. Consequences for management are applicable, for example, when trying to impose behaviours through learning (scarecrow tactics) where more experienced individuals will adjust their behaviour to the perceived risk. In such cases, variability in risk could be effective because even a low real risk can induce learning and avoidance behaviour.

## Supporting information

S1 Table Elk hunting times and types. (DOCX)

S2 Table Female elk sample size overview. (DOCX)

S3 Table Parameters estimated via model averaging of top ranked models selected in Table 2. (DOCX)

S1 Fig Map of the study area with female elk satellite relocations, and distribution of wildlife management units and protected areas. (DOCX)

S2 Fig Residuals’ variograms for top-ranked models. (DOCX)

## References

[1] Dingemanse NJ, Kazem AJN, Reale D, Wright J. Behavioural reaction norms: animal personality meets individual plasticity. Trends Ecol Evol. 2010;25(2):81–9. doi: 10.1016/j.tree.2009.07.013 19748700 10.1016/j.tree.2009.07.013

[2] Krebs JR, Davies NB. Behavioural ecology—An evolutionary approach. fourth edition ed: Blackwell publishing; 1996.

[3] Sih A, Bell A, Johnson JC. Behavioral syndromes: an ecological and evolutionary overview. Trends Ecol Evol. 2004;19(7):372–8. doi: 10.1016/j.tree.2004.04.009 16701288 10.1016/j.tree.2004.04.009

[4] Hellstrom G, Magnhagen C. The influence of experience on risk taking: results from a common-garden experiment on populations of Eurasian perch. Behav Ecol Sociobiol. 2011;65(10):1917–26.

[5] Dawson EH, Avargues-Weber A, Chittka L, Leadbeater E. Learning by observation emerges from simple associations in an insect model. Curr Biol. 2013;23(8):727–30. doi: 10.1016/j.cub.2013.03.035 23562271 10.1016/j.cub.2013.03.035

[6] Kitchen DM, Bergman TJ, Cheney DL, Nicholson JR, Seyfarth RM. Comparing responses of four ungulate species to playbacks of baboon alarm calls. Anim Cogn. 2010;13(6):861–70. doi: 10.1007/s10071-010-0334-9 20607576 10.1007/s10071-010-0334-9

[7] Mueller T, O'Hara RB, Converse SJ, Urbanek RP, Fagan WF. Social Learning of Migratory Performance. Science. 2013;341(6149):999–1002. doi: 10.1126/science.1237139 23990559 10.1126/science.1237139

[8] Knight RL, Gutzwiller KJ. Wildlife and recreationists. Washington, D.C.: Island Press; 1995.

[9] Ciuti S, Muhly TB, Paton DG, McDevitt AD, Musiani M, Boyce MS. Human selection of elk behavioural traits in a landscape of fear. Proc R Soc B-Biol Sci. 2012;279(1746):4407–16. 10.1098/rspb.2012.1483 PMC3479801 22951744

[10] Reale D, Festa-Bianchet M. Predator-induced natural selection on temperament in bighorn ewes. Anim Behav. 2003;65:463–70. 10.1006/anbe.2000.1530 11082229

[11] Lima SL, Bednekoff PA. Temporal variation in danger drives antipredator behavior: The predation risk allocation hypothesis. Am Nat. 1999;153(6):649–59. 10.1086/303202 29585647

[12] Sol D, Lapiedra O, Gonzalez-Lagos C. Behavioural adjustments for a life in the city. Anim Behav. 2013;85(5):1101–12.

[13] Wagner GP, Altenberg L. Perspective: Complex adaptations and the evolution of evolvability. Evolution. 1996;50(3):967–76. 28565291 10.1111/j.1558-5646.1996.tb02339.x

[14] Dall SRX, Houston AI, McNamara JM. The behavioural ecology of personality: consistent individual differences from an adaptive perspective. Ecol Lett. 2004;7(8):734–9.

[15] Carthey AJR, Banks PB. Naivete is not forever: responses of a vulnerable native rodent to its long term alien predators. Oikos. 2016;125(7):918–26.

[16] Swift KN, Marzluff JM. Wild American crows gather around their dead to learn about danger. Anim Behav. 2015;109:187–97.

[17] Griffin AS, Boyce HM. Indian mynahs, *Acridotheres tristis*, learn about dangerous places by observing the fate of others. Anim Behav. 2009;78(1):79–84.

[18] Knight RL, Temple SA. Origin of wildlife responses to recreationists In: Knight RL, Gutzwiller KJ, editors. Wildlife and Recreationists: Island Press; 1995 p. 81–91.

[19] Krantz GS. Brain size and hunting ability in earliest man. Curr Anthropol. 1968;9(5):450–63.

[20] Zedrosser A, Steyaert SMJG, Gossow H, Swenson JE. Brown bear conservation and the ghost of persecution past. Biol Conserv. 2011;144(9):2163–70.

[21] Brown JS, Vincent TL. Organization of predator-prey communities as an evolutionary game. Evolution. 1992;46(5):1269–83. 28569003 10.1111/j.1558-5646.1992.tb01123.x

[22] Ciuti S, Northrup JM, Muhly TB, Simi S, Musiani M, Pitt JA, et al Effects of humans on behaviour of wildlife exceed those of natural predators in a landscape of fear. PLoS One. 2012;7(11). 10.1371/journal.pone.0050611 PMC3509092 23226330

[23] Wright GJ, Peterson RO, Smith DW, Lemke TO. Selection of northern Yellowstone elk by gray wolves and hunters. J Wildlife Manage. 2006;70(4):1070–8.

[24] Burnham KP, Anderson DR, Huyvaert KP. AIC model selection and multimodel inference in behavioral ecology: some background, observations, and comparisons. Behav Ecol Sociobiol. 2011;65(1):23–35.

[25] Found R, St Clair CC. Behavioural syndromes predict loss of migration in wild elk. Anim Behav. 2016;115:35–46.

[26] Frair JL, Merrill EH, Beyer HL, Morales JM. Thresholds in landscape connectivity and mortality risks in response to growing road networks. Journal of Applied Ecology. 2008;45:1504–13.

[27] Morehouse AT, Boyce MS. From venison to beef: seasonal changes in wolf diet composition in a livestock grazing landscape. Frontiers in Ecology and the Environment. 2011;9:440–5.

[28] Muhly TB. Direct, indirect and predator-mediated effects of humans on a terrestrial food web: implications for conservation [PhD]. Calgary: University of Calgary; 2010.

[29] Muhly TB, Semeniuk C, Massolo A, Hickman L, Musiani M. Human activity helps prey win the predator-prey space race. PLoS One. 2011;6:e17050 doi: 10.1371/journal.pone.0017050 21399682 10.1371/journal.pone.0017050 PMC3047538

[30] ESRI. ArcGIS Desktop: Release 10. Redlands, CA: Environmental Systems Research Institute; 2011.

[31] Beyer HL. Geospatial Modelling Environment. 0.7.2 ed: Spatial Ecology LLC; 2012.

[32] R Development Core Team. R: A language and environment for statistical computing. Vienna, Austria: R Foundation for Statistical Computing; 2015.

[33] Ensing EP, Ciuti S, de Wijs FALM, Lentferink DH, ten Hoedt A, Boyce MS, et al GPS based daily activity patterns in European red deer and North American elk (*Cervus elaphus*): indication for a weak circadian clock in ungulates. PLoS One. 2014;9(9). 10.1371/journal.pone.0106997 PMC4160215 25208246

[34] Proffitt KM, Grigg JL, Garrott RA, Hamlin KL, Cunningham J, Gude JA, et al Changes in elk resource selection and distributions associated with a late-season elk hunt. J Wildlife Manage. 2010;74(2):210–8.

[35] Webb SL, Dzialak MR, Wondzell JJ, Harju SM, Hayden-Wing LD, Winstead JB. Survival and cause-specific mortality of female Rocky Mountain elk exposed to human activity. Popul Ecol. 2011;53(3):483–93.

[36] Lone K, Loe LE, Gobakken T, Linnell JDC, Odden J, Remmen J, et al Living and dying in a multi-predator landscape of fear: roe deer are squeezed by contrasting pattern of predation risk imposed by lynx and humans. Oikos. 2014;123(6):641–51.

[37] Benz RA, Boyce MS, Thurfjell H, Paton DG, Musiani M, Dormann CF, et al Dispersal Ecology Informs Design of Large-Scale Wildlife Corridors. PLoS One. 2016;11(9). 10.1371/journal.pone.0162989 PMC5033395 27657496

[38] Rivrud IM, Bischof R, Meisingset EL, Zimermann B, Loe LE, Mysterud A. Leave before it's too late: anthropogenic and environmental triggers of autumn migration in a hunted ungulate population. Ecology. 2016;97(4):1058–68. 28792596 10.1890/15-1191.1

[39] Singh NJ, Borger L, Dettki H, Bunnefeld N, Ericsson G. From migration to nomadism: movement variability in a northern ungulate across its latitudinal range. Ecol Appl. 2012;22(7):2007–20. 23210316 10.1890/12-0245.1

[40] Spiegelhalter DJ, Best NG, Carlin BR, van der Linde A. Bayesian measures of model complexity and fit. J Roy Stat Soc B. 2002;64:583–616.

[41] Vaida F, Blanchard S. Conditional Akaike information for mixed-effects models. Biometrika. 2005;92(2):351–70.

[42] Bates D, Maechler M, Bolker B, Walker S. Fitting Linear Mixed-Effects Models Using *lme4*. Journal of Statistical Software. 2015;67(1):1:48.

[43] Boyce MS, Pitt J, Northrup JM, Morehouse AT, Knopff KH, Cristescu B, et al Temporal autocorrelation functions for movement rates from global positioning system radiotelemetry data. Philos T R Soc B. 2010;365(1550):2213–9. 10.1098/rstb.2010.0080 PMC2894959 20566498

[44] Zuur AF, Ieno EN, Walker NJ, Saveliev AA, Smith GM. Mixed Effects Models and Extensions in Ecology with R. Gail M, Krickeberg K, Samet JM, Tsiatis A, Wong W, editors. New York: Springer Science+Business Media, LLC; 2009. 573 p.

[45] Bartoń K. MuMIn: Multi-model inference. R package version 1.15.6 ed2016.

[46] Proffitt KM, Gude JA, Hamlin KL, Messer MA. Effects of hunter access and habitat security on elk habitat selection in landscapes with a public and private land matrix. J Wildlife Manage. 2013;77(3):514–24.

[47] Allendorf FW, Hard JJ. Human-induced evolution caused by unnatural selection through harvest of wild animals. P Natl Acad Sci USA. 2009;106:9987–94. 10.1073/pnas.0901069106 PMC2702803 19528656

[48] Laporte I, Muhly TB, Pitt JA, Alexander M, Musiani M. Effects of wolves on elk and cattle behaviors: implications for livestock production and wolf conservation. PLoS One. 2010;5(8):9. 10.1371/journal.pone.0011954 PMC2915913 20694139

[49] Frair JL, Merrill EH, Visscher DR, Fortin D, Beyer HL, Morales JM. Scales of movement by elk (*Cervus elaphus*) in response to heterogeneity in forage resources and predation risk. Landscape Ecology. 2005;20(3):273–87.

[50] Cleveland SM, Hebblewhite M, Thompson M, Henderson R. Linking elk movement and resource selection to hunting pressure in a heterogeneous landscape. Wildlife Society Bulletin. 2012;36(4):658–68.

[51] Weckerly FW, Kennedy ML, Stephenson SW. Hunter-effort-harvest-size relationships among hunt types of white-tailed deer. Wildlife Society Bulletin. 2005;33(4):1303–11.

[52] Millspaugh JJ, Brundige GC, Gitzen RA, Raedeke KJ. Elk and hunter space-use sharing in South Dakota. J Wildlife Manage. 2000;64(4):994–1003.

[53] Sommer-Trembo C, Zimmer C, Jourdan J, Bierbach D, Plath M. Predator experience homogenizes consistent individual differences in predator avoidance. J Ethol. 2016;34(2):155–65.

[54] Blumstein DT, Evans CS, Daniel JC. An experimental study of behavioural group size effects in tammar wallabies, *Macropus eugenii*. Anim Behav. 1999;58:351–60. doi: 10.1006/anbe.1999.1156 10458887 10.1006/anbe.1999.1156

[55] Spiegel O, Leu ST, Bull CM, Sih A. What's your move? Movement as a link between personality and spatial dynamics in animal populations. Ecol Lett. 2017;20(1):3–18. doi: 10.1111/ele.12708 28000433 10.1111/ele.12708

[56] Kareklas K, Arnott G, Elwood RW, Holland RA. Plasticity varies with boldness in a weakly-electric fish. Front Zool. 2016;13. 10.1186/s12983-016-0154-0 PMC4895794 27274354

[57] Bonnot N, Verheyden H, Blanchard P, Cote J, Debeffe L, Cargnelutti B, et al Interindividual variability in habitat use: evidence for a risk management syndrome in roe deer? Behav Ecol. 2015;26(1):105–14.

[58] Archard GA, Braithwaite VA. The importance of wild populations in studies of animal temperament. J Zool. 2010;281(3):149–60.

[59] Manassa RP, McCormick MI, Dixson DL, Ferrari MCO, Chivers DP. Social learning of predators by coral reef fish: does observer number influence acquisition of information? Behav Ecol Sociobiol. 2014;68(8):1237–44.

[60] Manassa RP, McCormick MI. Social learning and acquired recognition of a predator by a marine fish. Anim Cogn. 2012;15(4):559–65. doi: 10.1007/s10071-012-0484-z 22453926 10.1007/s10071-012-0484-z

[61] Manassa RP, McCormick MI. Social learning improves survivorship at a life-history transition. Oecologia. 2013;171(4):845–52. doi: 10.1007/s00442-012-2458-x 22976775 10.1007/s00442-012-2458-x

[62] Manassa RP, McCormick MI, Chivers DP, Ferrari MCO. Social learning of predators in the dark: understanding the role of visual, chemical and mechanical information. Proc R Soc B-Biol Sci. 2013;280(1765). 10.1098/rspb.2013.0720 PMC3712441 23804616

[63] Hansen BB, Aanes R. Habituation to humans in a predator-free wild ungulate. Polar Biol. 2015;38(2):145–51.

[64] Bleicher SS, Brown JS, Embar K, Kotler BP. Novel predator recognition by Allenby's gerbil (*Gerbillus andersoni allenbyi*): do gerbils learn to respond to a snake that can "see" in the dark? Isr J Ecol Evol. 2016;62(3–4):178–85.

[65] Wolf M, McNamara JM. On the Evolution of Personalities via Frequency-Dependent Selection. Am Nat. 2012;179(6):679–92. doi: 10.1086/665656 22617258 10.1086/665656

[66] Wolf M, van Doorn GS, Leimar O, Weissing FJ. Evolution of animal personalities—Reply. Nature. 2007;450(7167):E5–E6. doi: 10.1038/nature06326 17538618 10.1038/nature05835

[67] Wolf M, van Doorn GS, Leimar O, Weissing FJ. Life-history trade-offs favour the evolution of animal personalities. Nature. 2007;447(7144):581–4. doi: 10.1038/nature05835 17538618 10.1038/nature05835

[68] Wolf M, Weissing FJ. Animal personalities: consequences for ecology and evolution. Trends Ecol Evol. 2012;27(8):452–61. doi: 10.1016/j.tree.2012.05.001 22727728 10.1016/j.tree.2012.05.001

[69] Sand H, Wikenros C, Wabakken P, Liberg O. Cross-continental differences in patterns of predation: will naive moose in Scandinavia ever learn? Proc R Soc B-Biol Sci. 2006;273(1592):1421–7. 10.1098/rspb.2005.3447 PMC1560300 16777732

[70] Tortosa FS, Barrio IC, Carthey AJR, Banks PB. No longer naive? Generalized responses of rabbits to marsupial predators in Australia. Behav Ecol Sociobiol. 2015;69(10):1649–55.

[71] Fey K, Banks PB, Ylonen H, Korpimaki E. Behavioural responses of voles to simulated risk of predation by a native and an alien mustelid: an odour manipulation experiment. Wildl Res. 2010;37(4):273–82.

[72] Stankowich T. Ungulate flight responses to human disturbance: A review and meta-analysis. Biol Conserv. 2008;141(9):2159–73.

