# Supplementary material for: Learning from the mistakes of others: How female elk (Cervus elaphus) adjust behaviour with age to avoid hunters
Source: PLoS One. 2017 Jun 14;12(6):e0178082. doi: 10.1371/journal.pone.0178082 (PMC5470680; doi:10.1371/journal.pone.0178082)
Supplement: S1 Table — (DOCX) [file pone.0178082.s001.docx]

**S1 Table -** Elk hunting times (start and end dates, MMDD) and types (bow, rifle) in the Wildlife Management Units (WMUs) of SW Alberta and SE British Columbia, Canada (2007 – 2012). Hunting was not allowed in the Waterton Lakes National Park.

|  | **Bow** | | **Rifle** | |  |
| --- | --- | --- | --- | --- | --- |
| **WMU** | Start | End | Start | End | Year |
| **4-1** | 9-1 | 9-9 | 9-10 | 10-20 | 2007 |
| **4-1** | 9-1 | 9-9 | 9-10 | 10-20 | 2008 |
| **4-1** | 9-1 | 9-9 | 9-10 | 10-20 | 2009 |
| **4-1** | 9-1 | 9-9 | 9-10 | 10-20 | 2010 |
| **4-1** | 9-1 | 9-9 | 9-10 | 10-20 | 2011 |
| **4-1** | 9-1 | 9-9 | 9-10 | 10-20 | 2012 |
| **300** | 9-5 | 10-24 | 10-25 | 12-20 | 2007 |
| **300** | 9-3 | 10-24 | 10-25 | 12-24 | 2008 |
| **302** | 9-5 | 10-24 | 10-25 | 12-20 | 2007 |
| **302** | 9-3 | 10-24 | 10-25 | 12-20 | 2008 |
| **302** | 9-9 | 10-24 | 10-25 | 12-20 | 2009 |
| **302** | 9-8 | 10-24 | 10-25 | 12-20 | 2010 |
| **302** | 9-1 | 10-24 | 10-25 | 12-20 | 2011 |
| **303** | 9-3 | 10-24 | 10-25 | 12-20 | 2008 |
| **303** | 9-9 | 10-24 | 10-25 | 12-20 | 2009 |
| **303** | 9-8 | 10-24 | 10-25 | 12-20 | 2010 |
| **303** | 9-1 | 10-24 | 10-25 | 12-20 | 2011 |
| **304** | 9-8 | 10-24 | 10-25 | 12-20 | 2010 |
| **304** | 9-1 | 10-24 | 10-25 | 12-20 | 2011 |
| **304** | 9-1 | 10-24 | 10-25 | 12-20 | 2012 |
| **305** | 9-9 | 10-24 | 10-25 | 12-20 | 2009 |
| **305** | 9-8 | 10-24 | 10-25 | 12-20 | 2010 |
| **305** | 9-1 | 10-24 | 10-25 | 12-20 | 2011 |
| **305** | 9-1 | 10-24 | 10-25 | 12-20 | 2012 |
| **306** | 9-9 | 10-24 | 10-25 | 12-20 | 2009 |
| **306** | 9-8 | 10-24 | 10-25 | 12-20 | 2010 |
| **306** | 9-1 | 10-24 | 10-25 | 12-20 | 2011 |
| **306** | 9-1 | 10-24 | 10-25 | 12-20 | 2012 |
| **308** | 9-8 | 10-24 | 10-25 | 12-20 | 2010 |
| **308** | 9-1 | 10-24 | 10-25 | 12-20 | 2011 |
| **308** | 9-1 | 10-24 | 10-25 | 12-20 | 2012 |
| **400** | 9-3 | 9-16 | 9-17 | 11-30 | 2007 |
| **400** | 9-5 | 9-16 | 9-17 | 11-30 | 2008 |
| **400** | 9-9 | 9-16 | 9-17 | 11-30 | 2009 |
| **400** | 9-8 | 9-16 | 9-17 | 11-30 | 2010 |
| **400** | 9-1 | 9-16 | 9-17 | 11-30 | 2011 |
| **402** | 9-9 | 9-16 | 9-17 | 11-30 | 2009 |
| **402** | 9-8 | 9-16 | 9-17 | 11-30 | 2010 |
| **402** | 9-1 | 9-16 | 9-17 | 11-30 | 2011 |
| **402** | 9-1 | 9-16 | 9-17 | 11-30 | 2012 |
| **4-23** | 9-1 | 9-9 | 9-10 | 10-20 | 2007 |
| **4-23** | 9-1 | 9-9 | 9-10 | 10-20 | 2008 |
| **4-23** | 9-1 | 9-9 | 9-10 | 10-20 | 2009 |
| **4-23** | 9-1 | 9-9 | 9-10 | 10-20 | 2010 |
| **4-23** | 9-1 | 9-9 | 9-10 | 10-20 | 2011 |
| **4-23** | 9-1 | 9-9 | 9-10 | 10-20 | 2012 |
|  |  |  |  |  |  |
|  |  |  |  |  |  |

Data sources:

- *Alberta Environment and Sustainable Resource Development AESRD official data*
- *British Columbia province official data*
