# Supplementary material for: Learning from the mistakes of others: How female elk (Cervus elaphus) adjust behaviour with age to avoid hunters
Source: PLoS One. 2017 Jun 14;12(6):e0178082. doi: 10.1371/journal.pone.0178082 (PMC5470680; doi:10.1371/journal.pone.0178082)
Supplement: S2 Table — (DOCX) [file pone.0178082.s002.docx]

**S2 Table** - Female elk monitored with satellite telemetry in SW Alberta and SE British Columbia (2007-2012). Step-lengths were computed for individuals monitored for 2 consecutive years, when relocations were collected every 2 hours. Step-lengths were not considered after the second year of monitoring because of rarefied sampling regimes (4-h fix rate). However, the use of forest and terrain ruggedness was computed over the entire monitoring period for each animal (ranging from 2 to 5 consecutive years of monitoring per individual elk).

| **#** | **Elk ID** | **Age at capture** | **Elk age (y.o.) during the monitoring period**  **(range shown: age recorded at capture – age during the last monitoring year)** | **Step-lengths recorded during 1^st^ year of monitoring** | **Step-lengths recorded during 2^nd^ year of monitoring** |
| --- | --- | --- | --- | --- | --- |
| 1 | 1 | 8 | 8-12 | 4139 | 4315 |
| 2 | 4 | 1 | 1-4 | 4079 | 4261 |
| 3 | 5 | 13 | 13-17 | 3893 | 4082 |
| 4 | 9 | 4 | 4-7 | 4040 | 4213 |
| 5 | 16 | 1 | 1-4 | 4045 | 4228 |
| 6 | 17 | 15 | 15-18 | 3826 | 4122 |
| 7 | 18 | 9 | 9-12 | 3874 | 4100 |
| 8 | 19 | 11 | 11-13 | 4138 | 3314 |
| 9 | 21 | 1 | 1-5 | 4066 | 4298 |
| 10 | 23 | 11 | 11-14 | 4114 | 4292 |
| 11 | 26 | 2 | 2-4 | 4202 | 3088 |
| 12 | 38 | 8 | 8-11 | 3699 | 3924 |
| 13 | 40 | 4 | 4-7 | 4003 | 4213 |
| 14 | 43 | 14 | 14-16 | 4045 | 3775 |
| 15 | 44 | 7 | 7-12 | 4123 | 4337 |
| 16 | 49 | 5 | 5-7 | 4166 | 3321 |
| 17 | 52 | 14 | 14-17 | 3518 | 3723 |
| 18 | 53 | 4 | 4-6 | 3608 | 3234 |
| 19 | 59 | 7 | 7-10 | 3510 | 3583 |
| 20 | 60 | 1 | 1-4 | 3570 | 3945 |
| 21 | 61 | 4 | 4-6 | 3208 | 3260 |
| 22 | 62 | 14 | 14-17 | 3439 | 3550 |
| 23 | 74 | 4 | 4-8 | 1710 | 2024 |
| 24 | 75 | 4 | 4-8 | 3422 | 3970 |
| 25 | 77 | 16 | 16-18 | 3678 | 3318 |
| 26 | 83 | 4 | 4-7 | 1537 | 1805 |
| 27 | 87 | 6 | 6-8 | 3023 | 3166 |
| 28 | 94 | 7 | 7-10 | 3123 | 4196 |
| 29 | 97 | 4 | 4-7 | 2758 | 3300 |
| 30 | 98 | 5 | 5-8 | 3191 | 4061 |
| 31 | 99 | 2 | 2-5 | 2210 | 2136 |
| 32 | 100 | 1 | 1-4 | 3160 | 3914 |
| 33 | 107 | 7 | 7-9 | 3141 | 3531 |
| 34 | 109 | 9 | 9-12 | 3074 | 4082 |
| 35 | 110 | 7 | 7-10 | 3136 | 4162 |
| 36 | 113 | 4 | 4-7 | 2986 | 3976 |
| 37 | 114 | 10 | 10-13 | 3245 | 4145 |
| 38 | 116 | 10 | 10-13 | 3320 | 4336 |
| 39 | 118 | 18 | 18-20 | 2947 | 3511 |
| 40 | 120 | 9 | 9-12 | 2669 | 3737 |
| 41 | 121 | 4 | 4-7 | 3443 | 3730 |
| 42 | 122 | 9 | 9-12 | 2582 | 3600 |
| 43 | 124 | 4 | 4-7 | 3024 | 3933 |
| 44 | 137 | 3 | 3-6 | 2889 | 3922 |
| 45 | 138 | 6 | 6-8 | 2839 | 3461 |
| 46 | 139 | 2 | 2-5 | 2748 | 3837 |
| 47 | 144 | 12 | 12-14 | 4111 | 5283 |
| 48 | 146 | 6 | 6-8 | 4131 | 2919 |
| 49 | 160 | 2 | 2-4 | 3353 | 3765 |
