# Supplementary material for: Learning from the mistakes of others: How female elk (Cervus elaphus) adjust behaviour with age to avoid hunters
Source: PLoS One. 2017 Jun 14;12(6):e0178082. doi: 10.1371/journal.pone.0178082 (PMC5470680; doi:10.1371/journal.pone.0178082)
Supplement: S3 Table — (DOCX) [file pone.0178082.s003.docx]

**S3 Table.** Parameters estimated via model averaging of top ranked models (Akaike weigths > 0.90) selected in Table 2, refitted using Restricted Estimate of Maximum Likelihood. All models have a random slope for true age and a random intercept for individual elk, as well as a random intercept for year. Reference levels for categorical predictors are: month [September], day of week [weekday], hunting season [no hunting], distance to road [>500m], and time of day [night]. Estimates reported in black are significant (p<0.05), whereas those reported in grey are not (based on whether 95% conditional Cis overlap zero).

| **Response variable:** | **Log step-length** | | **Use of terrain ruggedness** | | **Use of forest (0 = no forest, 1 = forest)** | |
| --- | --- | --- | --- | --- | --- | --- |
| **Fixed effect** | ***β*** | ***SE*** | ***β*** | ***SE*** | ***β*** | ***SE*** |
| Intercept | 4.165888 | 0.16325 | 18.170468 | 0.107463 | 0.128401 | 0.017676 |
| Month [October] | 0.080664 | 0.01363 | 0.274733 | 0.086852 | -0.318496 | 0.017104 |
| Month [November] | 0.007217 | 0.014167 | 2.425432 | 0.089199 | -0.243526 | 0.017581 |
| Month [December] | 0.040345 | 0.014326 | -1.258134 | 0.090318 | -0.737678 | 0.0177 |
| Canopy cover | 0.033161 | 0.005896 | 0.077496 | 0.035906 |  |  |
| Canopy cover^2 | 0.0028 | 0.009894 | 1.080948 | 0.062504 |  |  |
| Ruggedness | -0.1564 | 0.006415 |  |  | 0.136314 | 0.007198 |
| Ruggedness^2 | 0.040545 | 0.00377 |  |  | -0.160721 | 0.004828 |
| Day of week [Weekend] | 0.033162 | 0.010425 | 0.001756 | 0.067801 | 0.035571 | 0.013333 |
| Hunting season [Bow] | -0.04422 | 0.079134 | 9.687946 | 0.480114 | 0.280359 | 0.098411 |
| hunting season[Rifle] | 0.035109 | 0.039492 | 9.49973 | 0.214186 | -0.026074 | 0.042467 |
| Distance to road [close] | 0.023447 | 0.010513 | -3.962905 | 0.063352 | -0.123714 | 0.012664 |
| Time of day [Dawn] | 0.290475 | 0.013714 | 0.97799 | 0.090045 | 0.306603 | 0.017329 |
| Time of day [Day] | 0.161434 | 0.012368 | 1.556148 | 0.079415 | 1.10093 | 0.015412 |
| Time of day [Dusk] | 0.947659 | 0.013919 | 0.593675 | 0.090221 | 0.3147 | 0.017367 |
| Age | -1.12979 | 0.039204 | -0.111673 | 0.062564 | -0.016133 | 0.01223 |
| Age*Hunting season [Bow] | 0.409202 | 0.100347 | 5.163224 | 0.584232 | -0.552027 | 0.117604 |
| Age* Hunting season [Rifle] | 0.156269 | 0.049146 | 1.671345 | 0.261595 | -0.305249 | 0.051694 |
| Age* Distance to road [<500 m] | 0.025128 | 0.010005 | 0.155684 | 0.062643 | 0.295777 | 0.012435 |
| Age* time of day [Dawn] |  |  | 0.061407 | 0.089658 | -0.008146 | 0.017318 |
| Age*time of day [Day] |  |  | 0.01044 | 0.075964 | 0.027349 | 0.015444 |
| Age*time of day [Dusk] |  |  | 0.141776 | 0.089958 | 0.050338 | 0.017422 |
| Age* day of week [Weekend] | -0.00368 | 0.010421 | -0.104643 | 0.067686 | -0.016263 | 0.013406 |
|  |  |  |  |  |  |  |
