# Supplementary material for: Learning from the mistakes of others: How female elk (Cervus elaphus) adjust behaviour with age to avoid hunters
Source: PLoS One. 2017 Jun 14;12(6):e0178082. doi: 10.1371/journal.pone.0178082 (PMC5470680; doi:10.1371/journal.pone.0178082)

**S1 Fig.** – Map of the study area in southern Alberta (Wildlife management unit numbers with three digits) and British Columbia (Wildlife management unit numbers with a dash). Elk satellite relocations in gray. Background color represents the forest mask (green = forest, gray = no forest). White area at the bottom represents the USA.


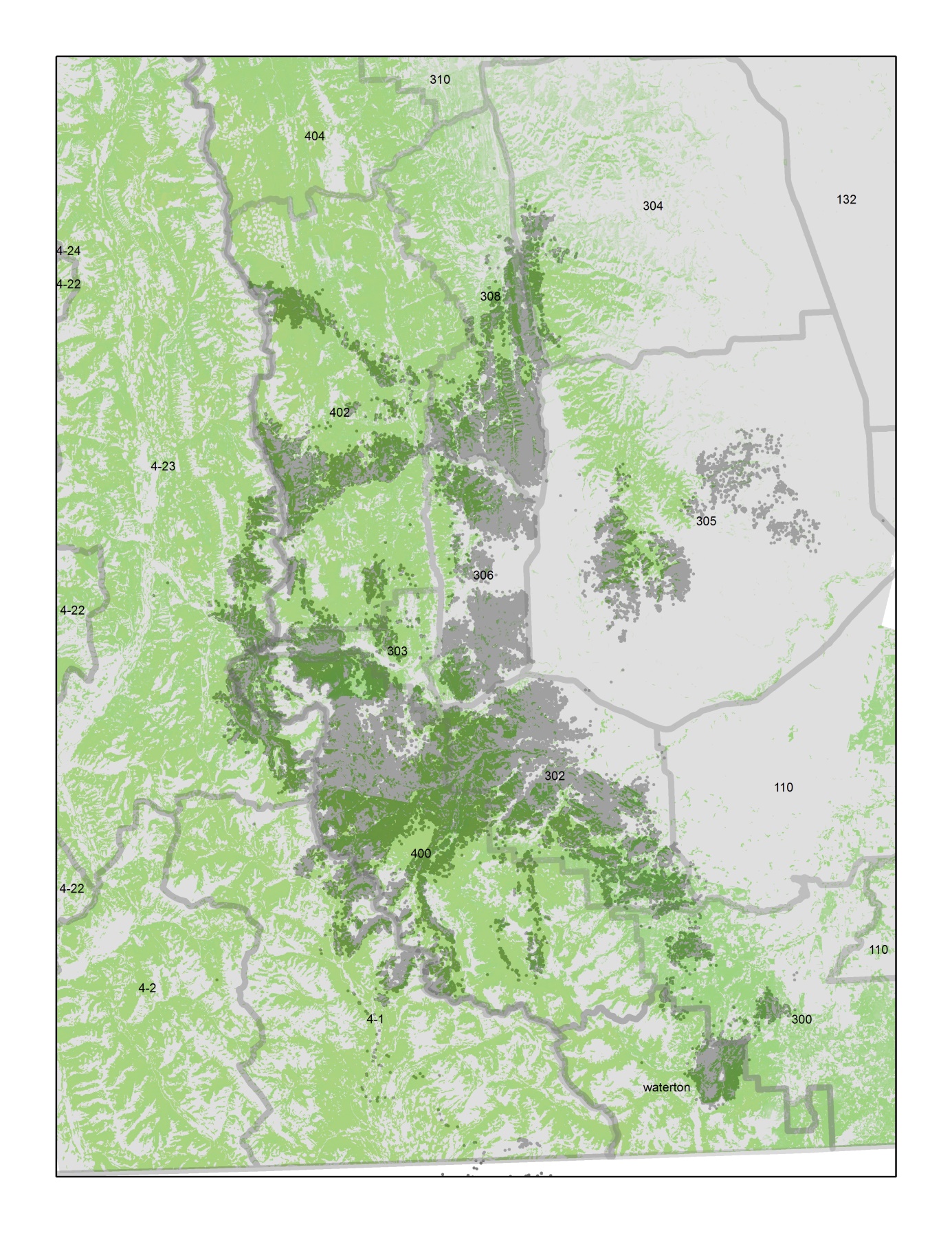

Supplement: S1 Fig — (DOCX) [file pone.0178082.s004.docx]
