# Supplementary material for: Learning from the mistakes of others: How female elk (Cervus elaphus) adjust behaviour with age to avoid hunters
Source: PLoS One. 2017 Jun 14;12(6):e0178082. doi: 10.1371/journal.pone.0178082 (PMC5470680; doi:10.1371/journal.pone.0178082)

**S2 Fig –** Residual semivariance as a function of distance (x-axes, in meters) for top-ranked models predicting step-length (left), use of terrain ruggedness (right), and use of forest (next page) by female elk in SW Alberta and SE British Columbia, Canada.


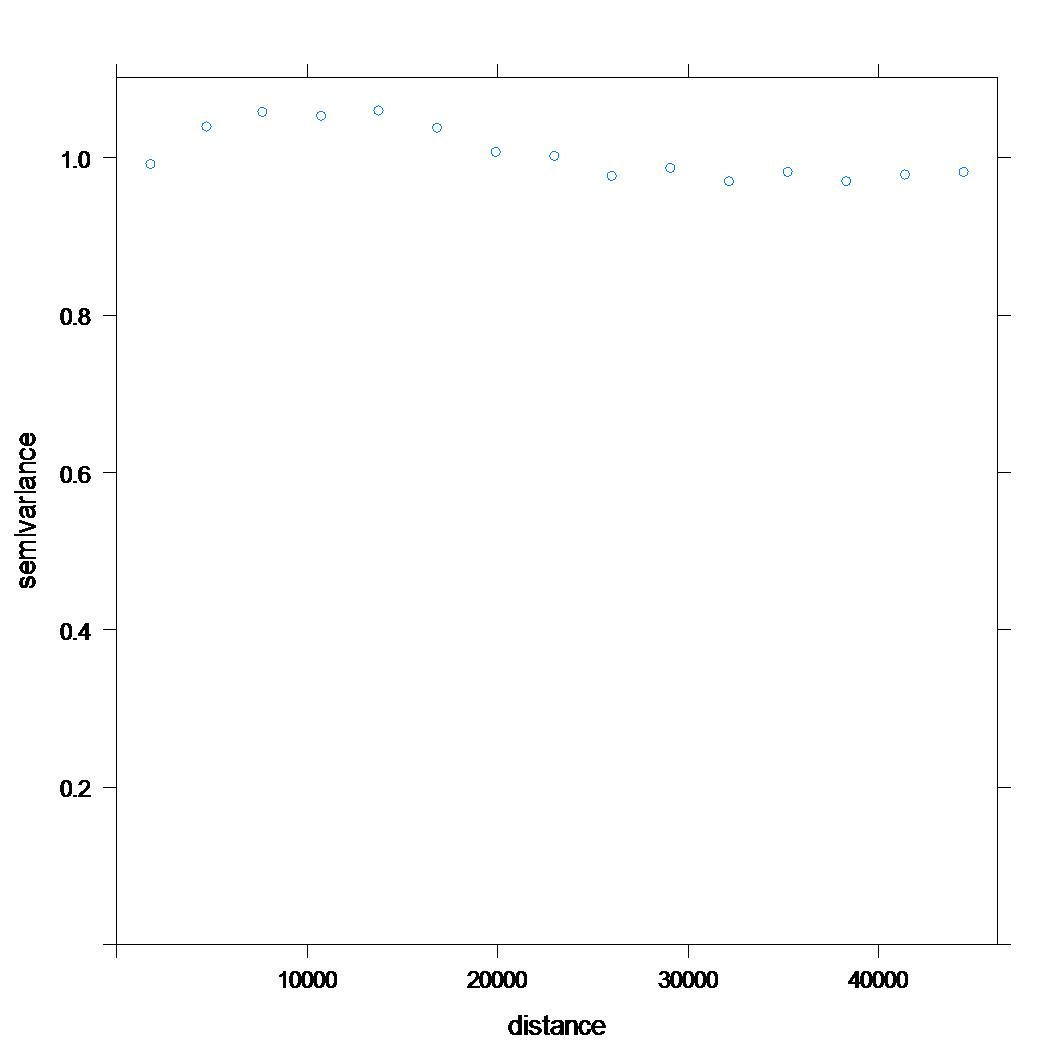

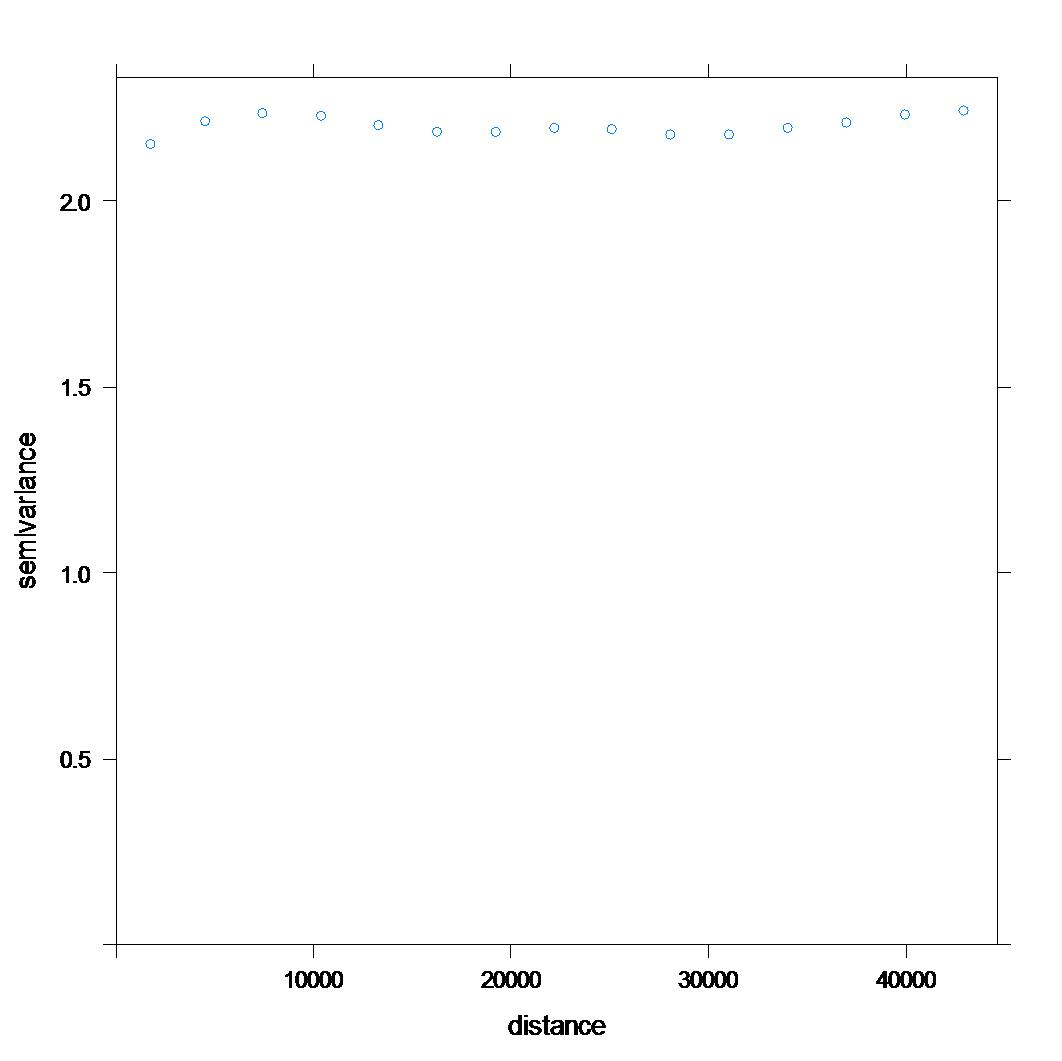


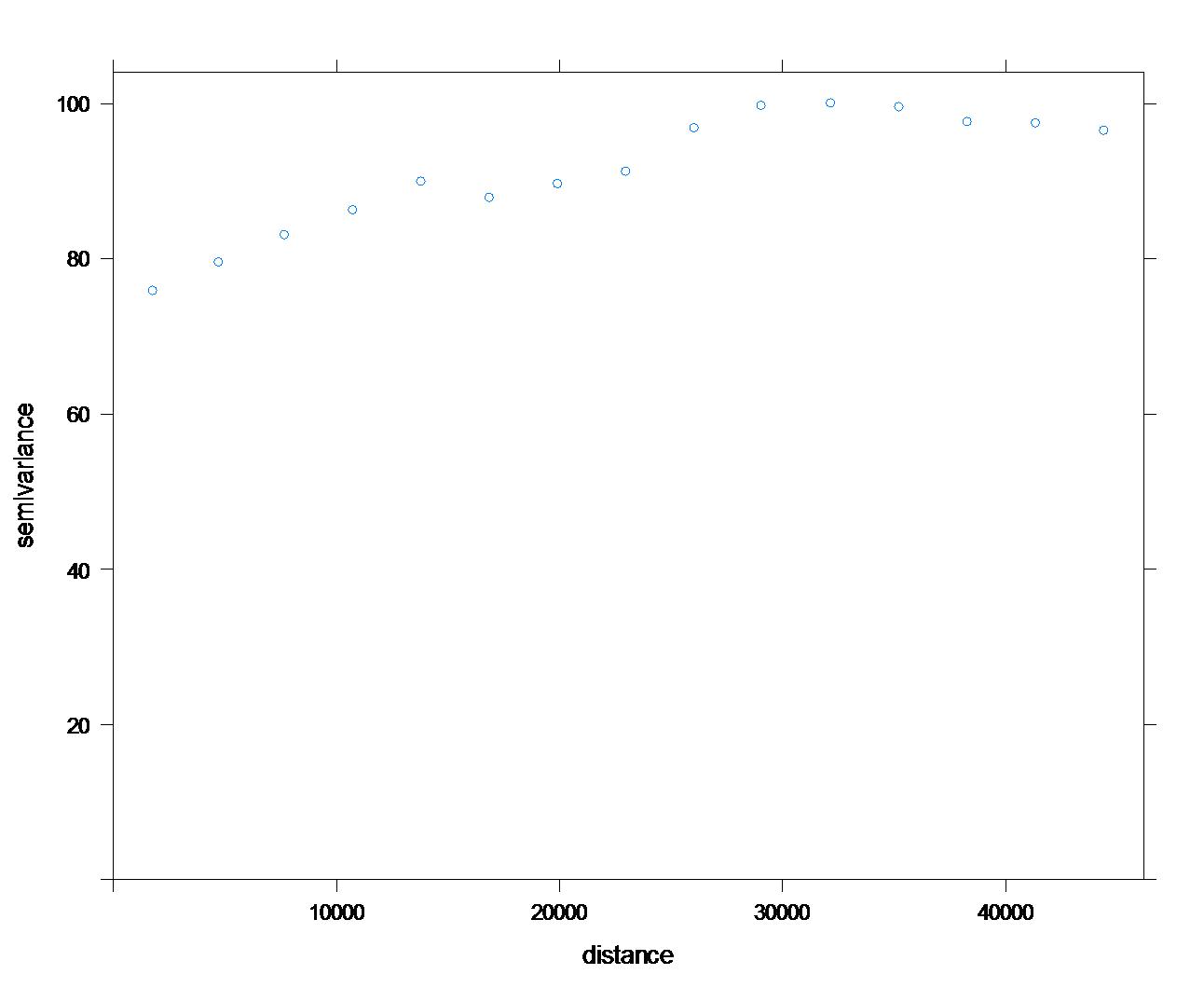

Supplement: S2 Fig — (DOCX) [file pone.0178082.s005.docx]
